# Translocator protein (18 kDa) (TSPO) is expressed in reactive retinal microglia and modulates microglial inflammation and phagocytosis

**DOI:** 10.1186/1742-2094-11-3

**Published:** 2014-01-08

**Authors:** Marcus Karlstetter, Caroline Nothdurfter, Alexander Aslanidis, Katharina Moeller, Felicitas Horn, Rebecca Scholz, Harald Neumann, Bernhard H F Weber, Rainer Rupprecht, Thomas Langmann

**Affiliations:** 1 Department of Ophthalmology, University of Cologne, D-50931 Cologne, Germany; 2 Department of Psychiatry and Psychotherapy, University of Regensburg, D-93053 Regensburg, Germany; 3 Institute of Reconstructive Neurobiology, University of Bonn, D-53127 Bonn, Germany; 4 Institute of Human Genetics, University of Regensburg, D-93053 Regensburg, Germany

**Keywords:** Translocator protein (18 kDa), Microglia, Retinal degeneration, Phagocytosis

## Abstract

**Background:**

The translocator protein (18 kDa) (TSPO) is a mitochondrial protein expressed on reactive glial cells and a biomarker for gliosis in the brain. TSPO ligands have been shown to reduce neuroinflammation in several mouse models of neurodegeneration. Here, we analyzed TSPO expression in mouse and human retinal microglia and studied the effects of the TSPO ligand XBD173 on microglial functions.

**Methods:**

TSPO protein analyses were performed in retinoschisin-deficient mouse retinas and human retinas. Lipopolysaccharide (LPS)-challenged BV-2 microglial cells were treated with XBD173 and TSPO shRNAs *in vitro* and pro-inflammatory markers were determined by qRT-PCR. The migration potential of microglia was determined with wound healing assays and the proliferation was studied with Fluorescence Activated Cell Sorting (FACS) analysis. Microglial neurotoxicity was estimated by nitrite measurement and quantification of caspase 3/7 levels in 661 W photoreceptors cultured in the presence of microglia-conditioned medium. The effects of XBD173 on filopodia formation and phagocytosis were analyzed in BV-2 cells and human induced pluripotent stem (iPS) cell-derived microglia (iPSdM). The morphology of microglia was quantified in mouse retinal explants treated with XBD173.

**Results:**

TSPO was strongly up-regulated in microglial cells of the dystrophic mouse retina and also co-localized with microglia in human retinas. Constitutive TSPO expression was high in the early postnatal Day 3 mouse retina and declined to low levels in the adult tissue. TSPO mRNA and protein were also strongly induced in LPS-challenged BV-2 microglia while the TSPO ligand XBD173 efficiently suppressed transcription of the pro-inflammatory marker genes chemokine (C-C motif) ligand 2 (CCL2), interleukin 6 (IL6) and inducible nitric oxide (NO)-synthase (iNOS). Moreover, treatment with XBD173 significantly reduced the migratory capacity and proliferation of microglia, their level of NO secretion and their neurotoxic activity on 661 W photoreceptor cells. Furthermore, XBD173 treatment of murine and human microglial cells promoted the formation of filopodia and increased their phagocytic capacity to ingest latex beads or photoreceptor debris. Finally, treatment with XBD173 reversed the amoeboid alerted phenotype of microglial cells in explanted organotypic mouse retinal cultures after challenge with LPS.

**Conclusions:**

These findings suggest that TSPO is highly expressed in reactive retinal microglia and a promising target to control microglial reactivity during retinal degeneration.

## Background

The translocator protein (18 kDa) (TSPO), previously known as the peripheral benzodiazepine receptor, is an integral part of the outer mitochondrial membrane [1] where it forms a complex with other mitochondrial proteins, such as the voltage-dependent anion channel (VDAC) and the adenine nucleotide transporter (ANT) [2]. TSPO mediates the transport of cholesterol into the inner mitochondrial membrane, where it serves as a precursor for steroids and neurosteroids [3]. Hence, the protein is constitutively expressed in steroidogenic tissues such as the adrenal gland, the gonads and the brain [4]. In the central nervous system TSPO is present both in neurons and activated glial cells [5-7]. Endogenous ligands of TSPO are cholesterol, porphyrins and active peptide fragments cleaved off from the diazepam binding inhibitor [8]. Glial up-regulation of TSPO is a major hallmark of neurodegenerative diseases [9] and various TSPO ligands have been developed as molecular markers to detect gliosis by means of Positron Emission Tomography (PET) imaging [10].

TSPO ligands are also under investigation as treatment options for a variety of neurological disorders, including Alzheimer’s disease [11], multiple sclerosis [12], neuropathic pain [13], peripheral nerve injury [9] and anxiety disorders [14]. Classical synthetic TSPO ligands, such as the benzodiazepine derivative 4′-chlorodiazepam (Ro5-4864) and the isoquinoline carboxamide PK11195, directly enhance GABAergic neurotransmission [15]. TSPO ligands such as etifoxine and XBD173 (emapunil) stimulate the synthesis of neurosteroids and may exert anti-inflammatory and neuroprotective effects [16].

Inherited retinal degenerations are clinically and genetically heterogeneous diseases characterized by progressive vision loss [17]. Although the individual mechanisms of pathogenesis remain to be resolved, microglial activation is a common hallmark of retinal degeneration [18]. The retinoschisin-deficient (Rs1h^-/Y^) mouse is a prototypic model for inherited retinal dystrophies with strong microglial reactivity [19,20]. Modulation of retinal microglia with docosahexaenoic acid could dampen microglial reactivity in Rs1h^-/Y^ mice and thereby reduced retinal degeneration [21]. TSPO ligands could potentially have a similar effect and may target the neurodegenerative cascade via their anti-inflammatory and microglia modulating effects.

In this study, we showed that TSPO expression is directly connected to retinal microgliosis in a mouse model of retinal degeneration and in human retinal sections. Moreover, we demonstrated that the TSPO ligand XBD173 induces an anti-inflammatory, neuroprotective and pro-phagocytic phenotype in microglia using cultures of murine and human microglial cell lines as well as mouse retinal explants.

## Methods

### Animals

MacGreen [22], Rs1h^-/Y^[19] and wild-type mice were all on a pure C57BL/ 6 J background. Animals were maintained in an air-conditioned environment on a 12-hour light–dark schedule at 22°C, and had free access to food and water. The health of the animals was regularly monitored, and all procedures complied with the German Law on Animal Protection and the Institute for Laboratory Animal Research Guide for the Care and Use of Laboratory Animals, 2011.

### Human tissue

Retinal samples of donors were obtained from the Eye Bank of the Center of Ophthalmology, University of Cologne, Germany. The donor age ranged between 54 and 72 years. Postmortem time ranged between 5 and 36 h. After dissection of the anterior segment, the remaining tissue included the posterior pole. The research followed the tenets of the Declaration of Helsinki.

### Reagents

*E. coli* 0111:B4 lipopolysaccharide and aminoglutethimide were purchased from Sigma Aldrich (St. Louis, MO, USA). XBD173 (emapunil) was obtained by custom synthesis from APAC Pharmaceuticals (Ellicott City, MD, USA). XBD173 was dissolved in ethanol.

### Cell culture and retinal explants

BV-2 microglia-like cells were cultured in RPMI/5% fetal calf serum (FCS) supplemented with 2 mM L-Glutamine and 195 nM β-mercaptoethanol. Isolation and culture of primary retinal microglia has been described previously [21]. BV-2 cells were stimulated with 50 ng/ml lipopolysaccharide (LPS) and various concentrations of XBD173 or ethanol as vehicle control. 661 W photoreceptor-like cells were a gift from Prof. Muayyad Al-Ubaidi (Department of Cell Biology, University of Oklahoma Health Sciences Center, Oklahoma City, OK, USA) and the culture conditions have been described elsewhere [23]. Human microglial cell lines (iPSdM) were generated from induced pluripotent stem (iPS) cell lines obtained by reprogramming from skin fibroblasts as previously described [24,25]. These cells proliferate without addition of growth factors and they were passaged 1:3 twice a week. The microglial phenotype was confirmed by flow cytometry (CD11b, CD16/32, CD36, CD45, CX3CR1). Retinas from MacGreen mice were rinsed in DMEM/Ham’s F12 medium supplemented with 1% FCS and placed on 25 mm circular Nucleopore filters (VWR, Darmstadt, Germany) with the photoreceptor side facing the membrane. After 24 h of *in vitro* culture with vehicle, 1 μg/ml LPS, 20 μM XBD173 or 1 μg/ml LPS + 20 μM XBD173, retinas were fixed and imaged in flat-mounts. Ramified and amoeboid microglial cells were directly imaged by green fluorescent protein (GFP) fluorescence using the Axioskop2 MOT Plus Apotome microscope (Carl Zeiss, Jena, Germany) and counted.

### Scratch wound-healing assay

A total of 400,000 BV-2 microglial cells were grown in six-well plates as 80% confluent monolayers and were wounded with a sterile 100 μl pipette tip. Thereafter, the cells were stimulated with 50 ng/ml LPS, 50 μM XBD173, 50 ng/ml LPS + 50 μM XBD173, or ethanol as solvent control. Migration into the open scar was documented with microphotographs taken at different time points after wounding using a Nikon ECLIPSE TE2000 inverted microscope (Nikon, Tokyo, Japan). The number of migrating cells was quantified by counting all cells within a 0.4 mm^2^ region in the center of each scratch. The number of migrated cells was then normalized to the average cell density to account for changes in proliferation. A minimum of five individual cultures was used to calculate the mean migratory capacity of each cell culture condition.

### Proliferation assay

For carboxyfluorescein diacetate succinimidyl ester (CFSE) proliferation assays, BV-2 microglial cells were labeled with 1 μM CFSE (e-Bioscience, San Diego, CA, USA) and cultured (1.5 × 10^5^ per well) in a six-well plate. After 24 h of culture with vehicle, 100 ng/ml LPS, 50 μM XBD173 or 100 ng/ml LPS + 50 μM XBD173, cells were stained with a fixable viability dye (e-Bioscience), to exclude dead cells from the analysis. The fluorescence intensity of CFSE-labeled BV-2 cells was analyzed by flow cytometry (FACS Canto II). Analysis of cell division was performed using FlowJo software (Treestar Inc., Ashland, OR, USA).

### shRNA knock-down of TSPO in BV-2 cells

For knockdown of endogenous TSPO in BV-2 cells, shRNA vectors were obtained from the RNAi consortium (TRC). Briefly, BV-2 cells were transfected with 2.5 μg vector DNA using TransIT®-LT1 transfection reagent (Mirus Bio LLC, Madison, WI, USA) to express TSPO-specific or scrambled shRNAs. Twenty-four hours after transfection cells were stimulated with vehicle, 50 ng/ml LPS, 20 μM XBD173 or 50 ng/ml LPS + 20 μM XBD173 for 12 hours before cells were harvested for RNA isolation and mRNA expression analysis.

### 661 W photoreceptor apoptosis assay

To test microglial neurotoxicity, a culture system of 661 W photoreceptors with microglia-conditioned medium was established. 661 W photoreceptor cells were incubated for 48 h either in their own medium or with culture supernatants from unstimulated, 50 ng/ml LPS, 50 μM XBD173 or 50 ng/ml LPS + 50 μM XBD173 treated microglial cells. The 661 W cell morphology was assessed by phase contrast microscopy and apoptotic cell death was determined with the Caspase-Glo® 3/7 Assay (Promega GmbH, Mannheim, Germany). Cells were lysed and incubated with a luminogenic caspase-3/7 substrate, which contains the tetrapeptide sequence DEVD. Luminescence was then generated by addition of recombinant luciferase and was proportional to the amount of caspase activity present. The luminescent signal was read on an Infinite F200 pro plate reader (Tecan, Crailsheim, Germany). A blank reaction was used to measure background luminescence associated with the cell culture system and Caspase-Glo® 3/7 Reagent (Promega). The value for the blank reaction was subtracted from all experimental values. Negative control reactions were performed to determine the basal caspase activity of 661 W cells. Relative luciferase units (RLU) reflect the level of apoptotic cell death in the different 661 W cell cultures.

### Nitrite measurement

Nitric oxide concentrations were determined by measuring the amount of nitrite produced by BV-2 microglial cells into the culture medium using the Griess reagent system (Promega). A 50 μl cell culture supernatant was collected and an equal volume of Griess reagent was added to each well. After incubation for 15 minutes at room temperature, the absorbance was read at 540 nm on an Infinite F200 pro plate reader (Tecan). The concentration of nitrite for each sample was calculated from a sodium nitrite standard curve.

### Phagocytosis assays

BV-2 microglial cells were pre-treated for 2 h with compounds before 4 μl latex bead solution (Polystyrene microparticles, Sigma Aldrich, St. Louis, MO, USA) was added to the wells. Cells were incubated for 6 h and five micrographs per well were taken using an AxioVert.A1 inverted microscope (Carl Zeiss). The phagocytic activity was determined by calculating the number of cells which phagocytosed 10 or more latex beads compared to all cells per field. The conditions for human microglial cells (iPSdM) were the same with the modification that cells were pre-treated for 24 h, the incubation time with beads was 24 h and only fully saturated cells were counted as positive. To study the microglial uptake of apoptotic photoreceptor cell material, 661 W photoreceptor cells were starved with serum deprivation, harvested and fluorescently labeled using CellTracker CM-DiI (Invitrogen, Carlsbad, CA, USA). For phagocytosis, BV-2 microglial cells were pre-treated for 2 h with compounds before 400 μl stained apoptotic 661 W solution was added for further 6 h. iPSdM cells were pre-treated for 24 h before 400 μl stained apoptotic 661 W solution was added for further 24 h. Cells were then fixed and nuclei were stained with 4′,6-diamidino-2-phenylindole. Fluorescence micrographs were taken and ImageJ software (National Institutes of Health, Bethesda, MD, USA) was used to determine the ratio of phagocytosed apoptotic photoreceptor fragments (red signal) relative to the total microglia cell number (DAPI signal).

### Phalloidin staining

BV-2 microglial cells or human microglial cells (iPSdM) were grown on cover slips in six-well plates and the indicated compounds were added for 24 h. Thereafter, the cells were fixed, permeabilized with 0.1% Triton X-100 and f-actin was fluorescently labeled using 0.1 μg/ml Phalloidin-TRITC (Sigma). The nuclei were stained using 4′,6-diamidino-2-phenylindole and photomicrographs were taken with an Axioskop2 MOT Plus Apotome microscope (Carl Zeiss).

### Immunohistochemistry

Immunohistochemical analyses were performed on 10 μm retinal sections embedded in optimal cutting temperature (OCT) compound (Hartenstein, Würzburg, Germany) or on retinal flat mounts. Samples were fixed in 4% paraformaldehyde, rinsed and rehydrated with PBS. Sections were blocked with a dried milk solution followed by an overnight incubation with primary antibodies at 4°C. Antibodies included rabbit anti-Iba1 antibody (Wako Chemicals, Neuss, Germany), rabbit anti-TSPO antibody (Abcam, Cambridge, UK), goat anti-MAP2 antibody (Santa Cruz Biotechnology, Santa Cruz, CA, USA), and goat anti-GFAP antibody (Santa Cruz Biotechnology). After washing, samples were labeled with a secondary antibody conjugated to Alexa488 (green) or Alexa594 (red) (Jackson Immuno-Research, West Grove, PA, USA) and counter-stained with DAPI. Sections and flat-mounts were mounted in DAKO fluorescent mounting medium (Dako Deutschland GmbH, Hamburg, Germany) and viewed with an Axioskop2 MOT Plus Apotome microscope (Carl Zeiss).

### Western blot analysis

Mouse retinal tissue was homogenized in cold RIPA buffer (20 mM Na-phosphate buffer, 150 mM NaCl, 5 mM EDTA, 1% Triton X-100 and protease inhibitors) using a TissueLyser LT (Qiagen, Hilden, Germany). Insoluble debris was removed by centrifugation for 15 minutes at 16,000 g. LPS-treated and control BV-2 microglia were directly lysed in RIPA buffer. Protein concentrations were determined by Bradford assay (Roti-quant, Roth, Karlsruhe, Germany). A total of 10 μg of microglial or 30 μg of total-retina proteins were separated by SDS-PAGE on 15% gels with PageRuler pre-stained protein ladder (Thermo Scientific, Waltham, MA, USA). Proteins were then transferred to 0.45 μm nitrocellulose membranes (Biorad, Munich, Germany). After blocking in TBS-T containing 5% nonfat dry milk, membranes were incubated with primary antibodies against TSPO (ab109497, Abcam,) or Actin (sc-1616, Santa Cruz Biotechnology). Blots were then incubated with secondary goat anti-rabbit IgG-HRP or rabbit anti-goat IgG-HRP antibodies (sc-2004, sc-2768, Santa Cruz Biotechnology). Enhanced chemiluminescence signals were then visualized and imaged with the MultiImage II system (Alpha Innotech, Santa Clara, CA, USA).

### RNA isolation and reverse transcription

Total RNA was extracted from total retina, BV-2 microglial cells or isolated retinal microglia according to the manufacturer’s instructions using the RNeasy Mini Kit (Qiagen). Purity and integrity of the RNA was assessed on the Agilent 2100 Bioanalyzer with the RNA 6000 Nano LabChip® reagent set (Agilent Technologies, Santa Clara, CA, USA). The RNA was quantified spectrophotometrically and then stored at −80°C. First-strand cDNA synthesis was performed with the RevertAid™ H Minus First Strand cDNA Synthesis Kit (Fermentas, Schwerte, Germany).

### Quantitative real-time RT-PCR

Amplifications of 50 ng cDNA were performed with an ABI7900HT machine (Applied Biosystems, Carlsbad, CA, USA) in 10 μl reaction mixtures containing 1 × TaqMan Universal PCR Master Mix (Applied Biosystems), 200 nM of primers and 0.25 μl of dual-labeled probe (Roche ProbeLibrary, Roche Applied Science, Basel, Switzerland). The reaction parameters were as follows: 2 minutes 50°C hold, 30 minutes 60°C hold and 5 minutes 95°C hold, followed by 45 cycles of 20 s 94°C melt and 1 minute 60°C anneal/extension. Primer sequences and Roche Library Probe numbers were as follows: CCL2, forward primer 5′-catccacgtgttggctca-3′, reverse primer 5′-gatcatcttgctggtgaatgagt-3′, probe #62; IL6, forward primer 5′-gatggatgctaccaaactggat-3′, reverse primer 5′-ccaggtagctatggtactccaga-3′, probe #6; iNOS, forward primer 5′-ctttgccacggacgagac-3′, reverse primer 5′- tcattgtactctgagggctga-3′, probe #13. Measurements were performed in triplicates and results were analyzed with an ABI sequence detector software version 2.3 using the ΔΔCt method for relative quantification.

### Pregnenolone ELISA

BV-2 cells were seeded on 24-well plates in 1 ml/well of RPMI/5% FCS supplemented with 2 mM L-Glutamine and 195 nM β-mercaptoethanol. After cells had attached after 6 h, 50 ng/ml LPS and/or 20 μM XBD173 were added to each well. After 21 h, cells were washed twice with HEPES assay buffer (140 mM NaCl, 5 mM KCl, 1.8 mM CaCl_2,_ 1 mM MgSO_4,_ 10 mM glucose, 10 mM HEPES/NaOH, pH 7.4) as described previously [26]. Then 1 ml of HEPES assay buffer supplemented with BSA (0.1%) and Trilostane (25 μM) (Sigma-Aldrich) was added to each well. Again 50 ng/ml LPS and/or 20 μM XBD173 were added. After 3 h the supernatants were removed to perform pregnenolone ELISA according to the manufacturer’s recommendations (IBL International, Hamburg, Germany). In brief, 50 μl of each sample were pipetted into a rabbit anti-pregnenolone antibody coated 96-microwell Plate. A total of 100 μl of pregnenolone-HRP conjugate was then added. Ready-to-use-calibrators were provided by IBL international. After 1 h of incubation and washing 150 μl of tetramethylbenzidine/hydrogen peroxide (TMB) substrate was added. Assays were read with a Tecan Spectra at 450 nm. Data were analyzed by Magellan Data Analysis Software (Tecan).

### Statistical analyses

Real-time quantitative RT-PCR data were analyzed with the ΔΔCt method using an unpaired Student’s *t*-test. Assays for nitrite secretion, microglial migration and pregnenolone ELISAs were analyzed with an unpaired Student’s *t* test. Caspase 3/7 assays and phagocytosis assays were analyzed with a Mann–Whitney *U*-test. *P* <0.05 was considered as statistically significant.

## Results

### Induction of TSPO expression in activated microglia of the retina

**Figure 1:**
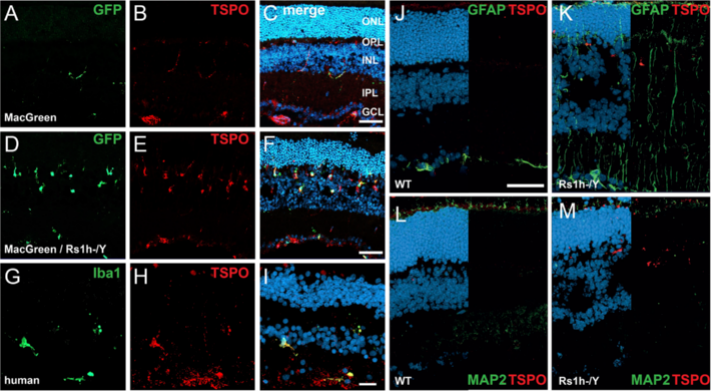
**TSPO expression as a marker for microgliosis in degenerating and aging retinas.** In MacGreen mice, representative photomicrographs show low TSPO expression in retinal microglia (green GFP signal and red TSPO immunofluorescence) and constitutive expression in astrocytes (red TSPO immunofluorescence) **(A-C)** but strong up-regulation in retinal microglia from MacGreen/Rs1h^-/Y^ mice **(D-F)**. The overlap of TSPO and Iba1 immunostaining also indicates co-expression in human retinal microglia **(G-I)**. TSPO immunostaining staining does not co-localize with the Müller cell marker GFAP **(J, K)** or the neuronal microtubule marker MAP2 **(L, M)**. ONL, outer nuclear layer; OPL, outer plexiform layer; INL, inner nuclear layer; IPL, inner plexiform layer; GCL, ganglion cell layer, GFP, green fluorescent protein; TSPO, translocator protein (18 kDa); GFAP, glial fibrillary acid protein; MAP2, microtubule-associated protein 2. Scale bar, 50 μm.

To identify genes regulated during activation of microglia, we have previously performed DNA microarray analysis of isolated retinal microglial cells from degenerating retinoschisin-deficient (Rs1h^-/Y^) and control macrophage/microglia MacGreen reporter mice [21]. Among the differently expressed transcripts, a significantly increased mRNA expression of TSPO was detected in activated retinal microglia (data not shown). Therefore, we determined the protein expression of TSPO in the retina and found a weak expression in the MacGreen reporter mouse, which was present in ramified microglia of the plexiform layers and astrocytes of the ganglion cell layer (Figure 1A-C). However, there was a remarkable increase of TSPO expression specifically in microglia of MacGreen/Rs1h^-/Y^ retinas (Figure 1D-F). Notably, there was nearly a full overlap of TSPO signals with amoeboid microglial cells which migrated to the degenerating inner nuclear layer (Figure 1F). In the human retina, TSPO expression was also evident in inner plexiform layer microglial cells as demonstrated by Iba1 co-immunostaining as well as in astrocytes (Figure 1G-I). To exclude that TSPO is significantly expressed in Müller cells or retinal neurons, immunostainings for glial fibrillary acid protein (GFAP) and microtubule-associated protein 2 (MAP2) were performed. Neither wild-type retinas nor Rs1h^-/Y^ retinas displayed an overlap of TSPO signals with GFAP (Figure 1J, K) or MAP2 (Figure 1L, M), respectively.

**Figure 2:**
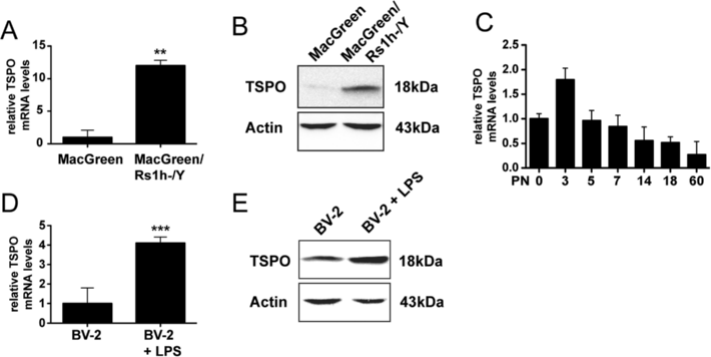
**TSPO mRNA and protein expression in reactive microglia. (A)** Strong induction of TSPO mRNA levels in isolated microglial cells from MacGreen/Rs1h^-/Y^ mice compared to MacGreen mice. **(B)** TSPO protein induction in total retinas from MacGreen/Rs1h^-/Y^ mice compared to MacGreen mice. **(C)** Temporal TSPO mRNA expression profiling shows a high early postnatal expression level and continuous decline to low levels in adult mouse retinas. **(D***,***E)** LPS activation of BV-2 microglial cells leads to the induction of TSPO transcripts **(D)** and protein **(E)**. Data show mean ± SD (n = 3/group, measured in triplicates) **P <0.01 MacGreen/Rs1h^-/Y^ versus MacGreen; ***P <0.001 BV-2 + 50 ng/ml LPS versus BV-2. LPS, lipopolysaccharide; TSPO, translocator protein (18 kDa).

To further verify the steep TSPO expression in reactive microglia, mRNA analysis of isolated retinal microglia from MacGreen and MacGreen/Rs1h^-/Y^ retinas was performed. There was a strong and significant increase of TSPO mRNA in MacGreen/Rs1h^-/Y^ microglia (12.01 ± 0.82, *P* <0.01) compared to MacGreen microglia (1.0 ± 1.07) (Figure 2A). TSPO induction was also confirmed on the protein level when total retinas of MacGreen/Rs1h^-/Y^ mice compared to MacGreen mice were analyzed (Figure 2B). We next performed TSPO transcript analysis in early postnatal development of the mouse retina. TSPO was present at higher levels in early retinal development and turned to lower levels in the adult retina (Figure 2C). We then analyzed whether the induction of TSPO expression is also present in cultured BV-2 microglia, which were activated by LPS. Treatment of BV-2 cells with 50 ng/ml LPS elicited a highly significant increase in TSPO levels (4.11 ± 0.31, *P* <0.001) compared to vehicle-treated BV-2 cells (1.0 ± 0.80) (Figure 2D). The increase of TSPO in LPS-activated BV-2 microglia was also confirmed on the protein level using Western blot analysis (Figure 2E).

### The selective TSPO ligand XBD173 dampens pro-inflammatory and neurotoxic microglial activation

**Figure 3:**
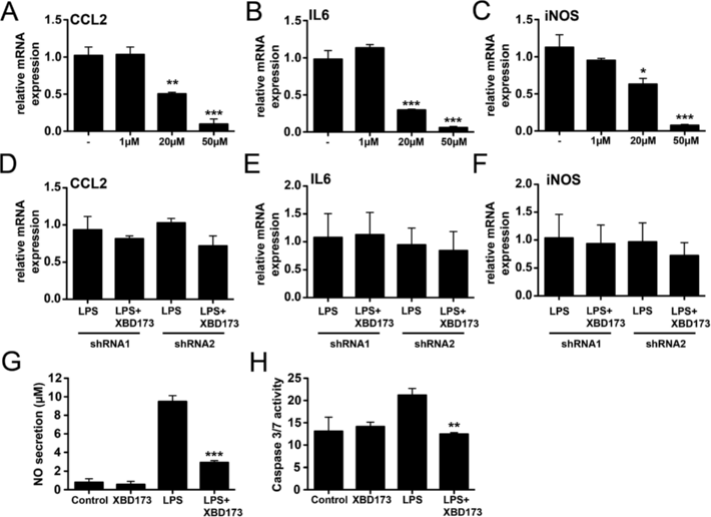
**The TSPO agonist XBD173 dampens gene transcription of pro-inflammatory markers and reduces microglial neurotoxicity.** LPS-activated BV-2 microglial cells were cultured in the presence of various concentrations of XBD173 for 24 h and the pro-inflammatory transcript markers CCL2 **(A)**, IL6 **(B)**, iNOS **(C)** were determined by *real time* qRT-PCR. Data show mean ± SD (n = 3/group, measured in triplicates) *P <0.05, **P <0.01, ***P <0.001 XBD173 + LPS versus LPS-treated cells. **(D-F)**, Knock-down of TSPO with two independent shRNAs abrogates the suppressing effects of XBD173 on CCL2 **(D)**, IL6 **(E)** and iNOS **(F)** gene expression in BV-2 cells. **(G)**, Production of NO as determined by detection of nitrite from BV-2 microglial cells treated with 50 μM XBD173 in the absence or presence of 50 ng/ml LPS. Data show mean ± SD (n = 9/group) ***P <0.001 XBD173 + LPS versus LPS-treated cells. **(H)**, 661 W photoreceptor cell cultures were treated with conditioned media from BV-2 microglial cells for 48 hours. The supernatant from control-stimulated, 20 μM XBD173-treated, 50 ng/ml LPS-treated, or 20 μM XBD173 + 50 ng/ml LPS-treated cells was added to 661 W photoreceptor cells and apoptosis-related caspase 3/7 activation was determined. Data show mean ± SD (n = 6/group) **P <0.01 XBD173 + LPS versus LPS-treated cells. CCL2, (C-C motif) ligand 2; IL6, interleukin-6; iNOS, inducible nitric oxide synthase; LPS, lipopolysaccharide; NO, nitric oxide.

We investigated whether stimulation of TSPO with the selective ligand XBD173 influences microglial reactivity. We first tested the mRNA expression of the chemo-attractant protein CCL2, the pro-inflammatory cytokine IL6 and iNOS in LPS-activated microglia upon treatment with three different doses of XBD173. The TSPO ligand significantly and dose-dependently suppressed mRNA levels of CCL2 (Figure 3A, *P* = 0.0014 for 20 μM XBD173 and *P* = 0.0003 for 50 μM XBD173 versus vehicle control), IL6 (Figure 3B, *P* = 0.0004 for 20 μM XBD173 and *P* = 0.0001 for 50 μM XBD173 versus vehicle control), and iNOS (Figure 3C, *P* = 0.0104 for 20 μM XBD173 and *P* = 0.0004 for 50 μM XBD173 versus vehicle control).

To test whether the anti-inflammatory effect of XBD173 depends on TSPO expression, shRNA mediated knock-down of TSPO was performed. Transfection of BV-2 cells with two different TSPO-specific shRNAs showed a 50 to 60% knock-down of TSPO mRNA levels compared to a scramble control (data not shown). The mRNA expression levels of CCL2 (Figure 3D), IL6 (Figure 3E) and iNOS (Figure 3F) were no longer suppressed by XBD173 treatment when either shRNA1 or shRNA2 that specifically target TSPO were present. This clearly implicates that the suppressing effect of XBD173 acts via TSPO in BV-2 cells.

When co-administered together with 50 ng/ml LPS, 50 μM XBD173 also strongly diminished NO-production from BV-2 microglial cells compared to vehicle-treated cells (Figure 3G, *P* <0.0001). We then performed an apoptosis assay of 661 W photoreceptor cells incubated in the presence of microglia-conditioned medium. LPS-activated microglial cells incubated in the presence of 50 μM XBD173 had a significantly lower pro-apoptotic effect on photoreceptor cells than microglia cultured with vehicle (Figure 3H, *P* <0.0022).

**Figure 4:**
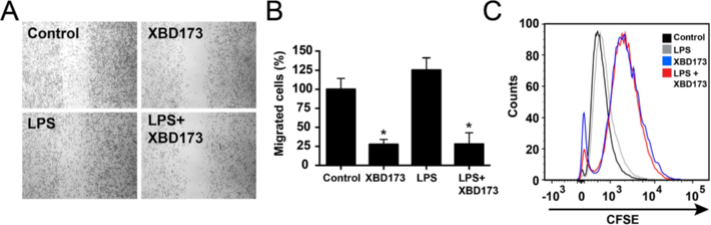
**The TSPO agonist XBD173 reduces microglial migration and proliferation. (A)** Scratch assay to mimic wound-healing in cultured BV-2 microglia treated with vehicle, 50 ng/ml LPS, 50 μM XBD173 or both. Microphotographs from scratched areas were quantified 8 h after treatment **(B)**. Data show mean ± SEM (n = 5/group) *P <0.05 XBD173 versus control, *P <0.001 XBD173 + LPS versus LPS-treated cells. **(C)**, CFSE-based proliferation assay of BV-2 microglial cells treated with vehicle, 100 ng/ml LPS, 50 μM XBD173 or 100 ng/ml LPS + 50 μM XBD173. The proliferation rate of BV-2 microglia was analyzed 24 hours after treatment using flow cytometry and a representative graph out of four repetitions is shown. CFSE, carboxyfluorescein diacetate succinimidyl ester; LPS, lipopolysaccharide; TSPO, translocator protein (18 kDa).

We then analyzed the effect of the TSPO ligand on microglial migration. Stimulation of BV-2 microglial cells with 50 μM XBD173 strongly reduced the migration of BV-2 cells in a wound-healing scratch assay (Figure 4A). This effect was quantified and significant in non-activated microglia (Figure 4B, *P* <0.0008) as well as LPS-preactivated BV-2 cells (Figure 4B, *P* <0.0406). We constantly noticed a reduced cell number in our culture assays and, therefore, tested a potential anti-mitotic effect of XBD173. CFSE labeling and FACS analyses clearly demonstrated that XBD173 reduced the proliferation rate of both unstimulated and LPS-treated BV-2 cells (Figure 4C).

### XBD173 increases filopodia formation and phagocytosis in murine and human microglia

**Figure 5:**
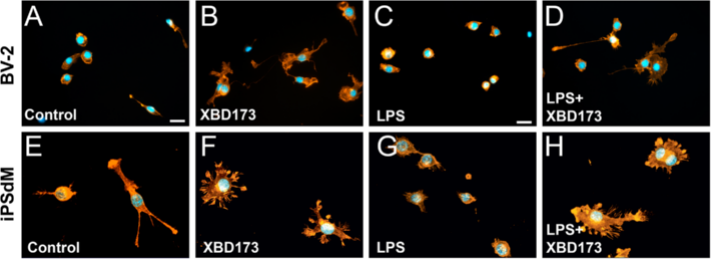
**The TSPO ligand XBD173 promotes microglial filopodia formation. (A-D)** Representative images of phalloidin-stained murine BV-2 microglial cells treated with 50 μM XBD173 in the absence or presence of 50 ng/ml LPS. **(E-H)** Representative images of phalloidin-stained human iPS-derived microglia (iPSdM) treated with 30 μM XBD173 in the absence or presence of 250 ng/ml LPS. LPS, lipopolysaccharide; TSPO, translocator protein (18 kDa).

To investigate further functional consequences of treatment with the TSPO ligand, we tested the effects of TSPO activation on the morphology and phagocytosis of murine BV-2 microglia and human microglial cells (iPSdM) derived from human induced pluripotent stem cells. Staining of the f-actin cytoskeleton with phalloidin indicated that XBD173 caused a prominent formation of filopodia in murine BV-2 cells either in the absence or presence of LPS (Figure 5A-D). This effect was even more pronounced in human iPSdM, which showed a large rim of flattened filopodia (Figure 5E-H).

**Figure 6:**
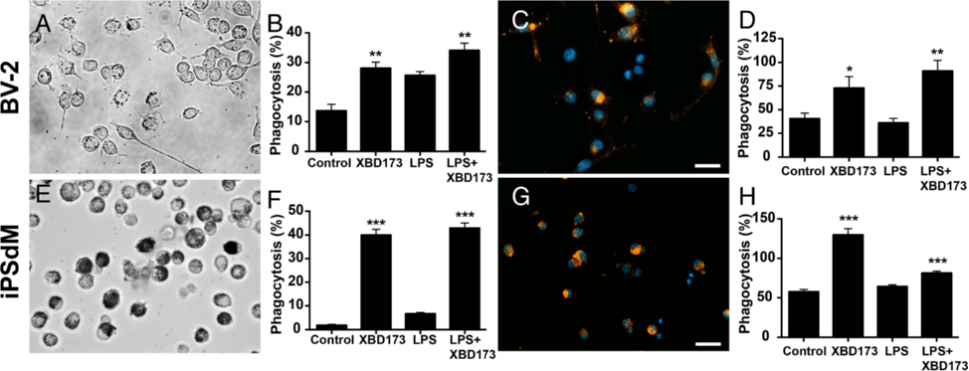
**The TSPO ligand XBD173 increases microglial phagocytosis.** Phagocytosis was monitored in BV-2 cells incubated with latex beads **(A, B)** or CM-DiI-stained apoptotic 661 W photoreceptor material **(C, D)**. Data show mean ± SEM (n = 9/group) **P <0.01 XBD173 + LPS versus LPS-treated cells, *P <0.05 XBD173 versus vehicle-treated cells. Phagocytosis was monitored in human iPSdM cells incubated with latex beads **(E, F)** or CM-DiI-stained apoptotic 661 W photoreceptor material **(G, H)**. Data show mean ± SEM (n = 9/group) ***P <0.01 XBD173 + LPS versus LPS-treated cell and XBD173 versus vehicle-treated cells. LPS, lipopolysaccharide; TSPO, translocator protein (18 kDa).

This striking phenomenon of XBD173-induced filopodia formation prompted us to analyze the phagocytic capacity in detail using latex beads and CM-DiI-stained apoptotic 661 W photoreceptor material as a more physiological trigger. BV-2 microglial cells stimulated with XBD173 showed a significantly higher phagocytosis rate of latex beads in non-activated cells (Figure 6A, B, *P* = 0.0013) as well as LPS-preactivated cells (Figure 6A, B, *P* = 0.0029). A similar effect of XBD173 was noticed with fluorescent apoptotic 661 W photoreceptors in non-activated (Figure 6C, D, *P* = 0.0192) and LPS-incubated BV-2 cells (Figure 6C, D, *P* = 0.0013). XBD173 had a similar stimulating effect on the phagocytic potential of human microglial cells with strongly increased uptake of latex beads (Figure 6E, F, *P* <0.0001) and CM-DiI-stained apoptotic 661 W membranes (Figure 6G, H, *P* <0.0001).

**Figure 7:**
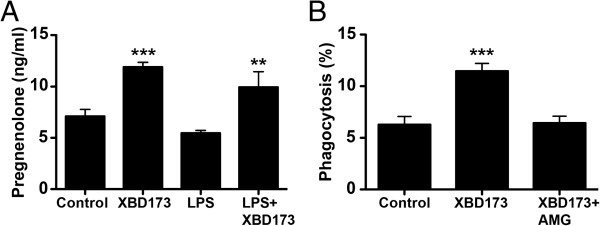
**The TSPO ligand XBD173 increases pregnenolone levels and CYP11A1 inhibition prevents XBD173-induced phagocytosis. (A)** Pregnenolone levels in cell culture supernatants of BV-2 cells treated with 20 μM XBD173, 50 ng/ml LPS, or 20 μM XBD173 + 50 ng/ml LPS for 24 hours. Data show mean ± SD (n = 3/group) ***P <0.001 XBD173 versus vehicle-treated cells, **P <0.01 XBD173 + LPS versus LPS-treated cells. **(B)** Quantification of latex bead phagocytosis of BV-2 cells treated with 20 μM XBD173 or 20 μM XBD173 + 100 μM aminoglutethimide for 24 hours. Data show mean ± SEM (n = 30/group) ***P <0.0001 XBD173 versus control cells. LPS, lipopolysaccharide; TSPO, translocator protein (18 kDa).

To test whether the phenomenon of XBD173-induced phagocytosis is dependent on neurosteroid synthesis, we first quantified pregnenolone levels in BV-2 cells. Stimulation of BV-2 microglia with either 20 μM XBD173 alone (Figure 7A, *P* <0.0005) or together with 50 ng/ml LPS (Figure 7A, *P* <0.0074) strongly increased pregnenolone levels measured in the cell culture supernatant. We next performed bead phagocytosis assays with BV-2 cells stimulated with 20 μM XBD173 in the presence or absence of the CYP11A1 inhibitor aminoglutethimide. As previously shown in Figure 6, BV-2 cells stimulated with XBD173 showed a higher phagocytosis potential than control cells (Figure 7B, *P* <0.0001). Interestingly, this effect was completely prevented when the cells were co-incubated with 20 μM XBD173 and 100 μM aminoglutethimide (Figure 7B). These data suggest that the phagocytosis promoting effect of XBD173 requires pregnenolone synthesis.

### XBD173 reduces the number of LPS-alerted amoeboid microglia in living retinal explants

**Figure 8:**
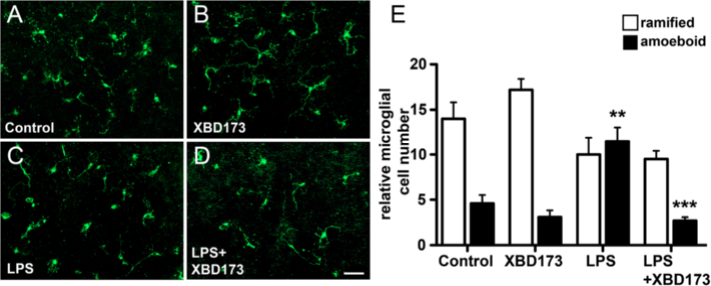
**The TSPO ligand XBD173 reduces the number of alerted amoeboid retinal microglia *ex vivo*. (A-D)** Representative GFP images of the retinal microglia network in explanted mouse retinas treated for 24 hours with vehicle **(A)**, 20 μM XBD173 **(B)**, 1 μg/ml LPS + vehicle **(C)** and 1 μg/ml LPS + 20 μM XBD173 **(D)**. **(E)** Quantification of ramified and amoeboid microglial cells in 10 independent image areas of two individual flat mounts (mean ± SEM). **P <0.01 for amoeboid cells in LPS versus control explants; ***P <0.0001, for amoeboid cells in XBD173 + LPS versus LPS-treated explants. GFP, green fluorescent protein; LPS, lipopolysaccharide; TSPO, translocator protein (18 kDa).

Finally, we analyzed whether the XBD173-dependent morphological transformation of microglial cells can also be observed in the *ex vivo* retina. Retinas from MacGreen reporter mice were used to enable easy GFP-based analysis of microglial ramification. Retinal explants cultured for 24 h *in vitro* retained their ramified morphology (Figure 8A) and the microglial network was not significantly affected by XBD173 alone (Figure 8B). In contrast, retinal microglia dramatically changed their morphology in the presence of LPS with a large fraction of bloated amoeboid cells (Figure 8C). This LPS-induced morphological transition of microglia was effectively suppressed by XBD173 (Figure 8D). We then performed a quantitative analysis of ramified and amoeboid microglial cells in all retinal explants. As already indicated in Figure 7A-D, the number of amoeboid cells was significantly increased in LPS-treated cultures compared to vehicle-treated explants (Figure 8E, *P* = 0.0012). Interestingly, co-incubation of explants with LPS and XBD173 resulted in a strongly reduced number of alerted amoeboid microglia cells (Figure 8E, *P* <0.0001). Thus, XBD173 was not only able to influence microglial reactivity *in vitro* but also significantly affected microglia in living mouse retinas.

## Discussion

Based on its selective expression in activated glial cells of the brain, TSPO is a marker for brain gliosis and TSPO ligands have been developed for *in vivo* imaging in human and mouse [27,28]. In the present study, we now show that selective up-regulation of TSPO is closely associated with the reactivity of microglia during retinal degeneration. To our knowledge, this is the first report to identify TSPO as a biomarker of activated microglia both in mouse and human retinal tissue. Major questions are why and how TSPO is up-regulated in reactive retinal microglia. Microgliosis in the retina of a mouse model of retinoschisin-deficiency starts at postnatal Day 14, peaks at P21 and then declines to lower levels [29]. The peak of microglial reactivity perfectly overlaps with high induction of TSPO expression levels. In the brain, TSPO detects activation of both microglia and astrocytes as a result of injury but also during recovery from injury [30,31], indicating that the presence of TSPO on activated glia may be a self-limiting mechanism of activation and proliferation. Our data of up-regulated TSPO expression in LPS-stimulated BV-2 microglia revealed that TLR4 signaling may play a crucial role in TSPO induction. In line with our experiments, up-regulation of TSPO in the recovery phase from neuropathic pain was prevented by pharmacological blockade of TLR4 [13]. We, therefore, hypothesize that retinal damage and the presence of damage-associated molecular patterns may trigger TLR4 signaling on microglia and thereby influence TSPO expression.

Our experiments demonstrate that the selective TSPO ligand XBD173 efficiently reversed the LPS-triggered production of the pro-inflammatory mediators CCL2, IL6 and iNOS. The TSPO ligand and mitochondrial effector PK11195 effectively inhibited LPS-induced microglial expression of COX-2 and TNF-alpha via modulation of Ca2^+^-mediated signaling pathways [32]. The same compound reduced the expression of pro-inflammatory cytokines and neuronal apoptosis in quinolinic-acid-treated rat brain [33]. These findings together with our data on the global influence of XBD173 on gene expression revealed that targeting TSPO has a broad influence on inflammatory signaling in microglia. As we have shown in microglial cell culture, one potential mechanism of the effects of XBD173 could be the synthesis of pregnenolone, as has been previously shown for astrocytes [34]. These findings support the concept that microglia locally synthesize the anti-inflammatory neurosteroid precursor pregnenolone after activation of TSPO by respective ligands.

Our studies revealed that TSPO significantly influenced the f-actin cytoskeleton and fostered the formation of filopodia along with prominent effects on microglial migration and phagocytosis. A high phagocytic activity with a low migratory capacity is a typical hallmark of homeostatic microglia which constantly survey their environment with long protrusions [35]. Over the last years it has also become clear that microglial phagocytosis of apoptotic cells is largely anti-inflammatory [36]. Thus, we hypothesize that induction of TSPO signaling may shift alerted microglia to a more homeostatic and less inflammatory state. In line with this, TSPO ligands have been shown to influence chemotaxis and phagocytosis in peripheral blood cells [37-39]. TSPO overexpression also increased the proliferation and migratory capacity of rat C6 glioma cells whereas treatment with the TSPO ligand PK11195 had a strong anti-proliferative effect and exerted pro-apoptotic activity on these cells [40]. Treatment of LPS-challenged microglia with XBD173 could effectively reduce the number of amoeboid cells in the explanted mouse retina but did not significantly increase the fraction of ramified cells. This clearly indicates that TSPO signaling may serve to control microglia dynamics during the activation and/or resolution phase of retinal damage. In the native retina, endogenous ligands for TSPO may fulfill this function. Several endogenous molecules that bind TSPO have been identified in steroidogenic tissues, including the protein diazepam binding inhibitor (DBI) [41]. DBI can be cleaved into several active peptide fragments such as octadecaneuropeptide (ODN) and trikontatetraneuropeptide (TTN) which are released from astrocytes [10]. TTN then stimulates neurosynthesis in C6 glioma cells by acting on TSPO [8]. Thus, we hypothesize that either retinal astrocytes or Müller cells may express DBI and secrete active peptides to control microglial activity via targeting of TSPO.

## Conclusions

We have shown that TSPO is highly expressed in reactive retinal microglia and BV-2 cells stimulated with LPS. The selective TSPO ligand XBD173 efficiently dampened pro-inflammatory gene expression in BV-2 microglial cells and reduced their neurotoxic potential on 661 W photoreceptor cells. XBD173 treatment of BV-2 cells and human iPS-derived microglial cells also promoted filopodia formation and phagocytic uptake. In the explanted mouse retina, XBD173 treatment blocked the LPS-dependent accumulation of amoeboid microglial cells. In conclusion, our data implicate that TSPO expression is connected to retinal microglia reactivity and that selective TSPO ligands may be a promising therapeutic approach to dampen microgliosis during retinal degeneration.

## Abbreviations

CCL2: chemokine (C-C motif) ligand 2; CFSE: carboxyfluorescein diacetate succinimidyl ester; DBI: diazepam binding inhibitor; FACS: Fluorescence Activated Cell Sorting; FCS: fetal calf serum; GFAP: glial fibrillary acid protein; IL: interleukin; iNOS: inducible nitric oxide synthase; iPS: induced pluripotent stem cell; iPSdM: induced pluripotent stem cell-derived microglia; LPS: lipopolysaccharide; MAP2: microtubule-associated protein 2; NO: nitric oxide; TSPO: translocator protein (18 kDa).

## Competing interests

RR has served as a consultant for Novartis developing TSPO ligands as anxiolytics and is a member of Novartis advisory boards. All other authors declare no competing financial interests.

## Authors’ contributions

TL, BW, HN and RR designed the research. MK, CN, AA, KM, FH, RS performed the research. MK, CN, HN and RS analyzed the data. TL and RR wrote the paper. All authors read and approved the final manuscript.
